# Spinocerebellar ataxia type 3/Machado-Joseph disease manifested as spastic paraplegia: A clinical and genetic study

**DOI:** 10.3892/etm.2014.2136

**Published:** 2014-12-16

**Authors:** YANMIN SONG, YUNHAI LIU, NING ZHANG, LILI LONG

**Affiliations:** Department of Neurology, Xiangya Hospital, Central South University, Changsha, Hunan 410008, P.R. China

**Keywords:** spastic paraplegia, hereditary spinocerebellar ataxia type 3, Machado-Joseph disease gene, mutation analysis, genetic anticipation, CAG trinucleotide repeats

## Abstract

The aim of the present study was to conduct a familial investigation and provide a genetic diagnosis to a family presenting with spastic paraplegia and clinically diagnosed with hereditary spastic paraplegia (HSP). Blood samples were obtained from the family, and mutations in the gene causing spinocerebellar ataxia type 3 (SCA3)/Machado-Joseph disease (MJD), known as MJD1, were analyzed using the polymerase chain reaction, 8% denaturing polyacrylamide gel electrophoresis, and T-vector ligation and sequencing. The trinucleotide repeat number of the mutant allele was 80, leading to a genetic diagnosis of SCA3/MJD. This suggests that patients with SCA3/MJD characteristically present with typical spastic paraplegia without evident manifestations of ataxia. For those families with HSP involving the nervous system and showing genetic anticipation, an MJD1 genetic diagnosis should be considered to assist in clinical diagnosis of HSP.

## Introduction

Hereditary spastic paraplegia (HSP) is a group of genetic diseases of the nervous system with clinical and genetic heterogeneity. Epidemiological studies have found that the prevalence of HSP is an estimated 1.27–12.1 cases per 100,000 individuals in Europe (1,2). This disease is manifested as a slowly progressive weakness of the lower extremities and spastic paraplegia. HSP can be divided into two types: The pure form and the complicated form. The pure form is only characterized by spastic paraplegia, i.e. progressive muscular hypertonia and weakness of the lower extremities (3,4). The complicated form is accompanied with extramedullary damage, such as mental retardation, extrapyramidal symptoms, ataxia, optic atrophy, retinal pigment degeneration, deafness, muscle atrophy and polyneuropathy (5). Hereditary spinocerebellar ataxia (SCA) is another group of genetic diseases involving the human nervous system. Spinocerebellar ataxia type 3 (SCA3)/Machado-Joseph disease (MJD), is the common subtype of SCA in mainland China (6). In addition, some cross symptoms are exhibited between HSP and SCA3/MJD. In the present study, a family showing genetic anticipation, spastic paraplegia and exophthalmos was investigated.

## Subjects and methods

### Subjects

**Figure 1:**
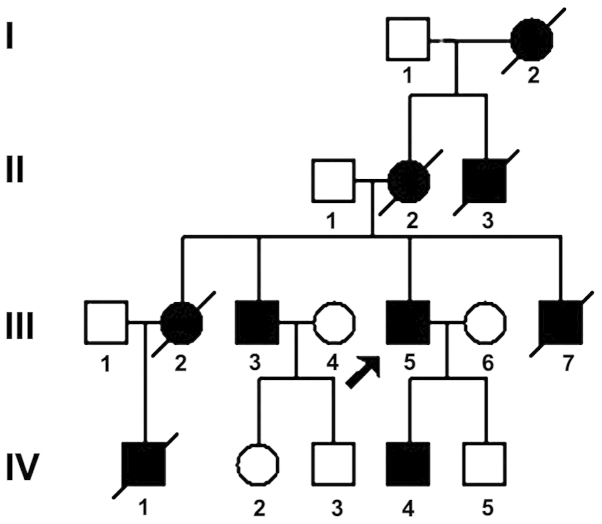
Pedigree of the family. Circles represent females, squares represent males; unfilled shapes represent unaffected family members, filled shapes represent affected family members, a line through a filled shape represents an affected, dead family member; and an arrow represents the proband.

The pedigree of a Han family from Hunan, China, in which four generations showed autosomal dominant inheritance, is illustrated in Fig. 1. A detailed inquiry was conducted into 17 individuals, six of whom have succumbed. The nine patients included six males and three females. The patients were diagnosed based on the Harding criteria (7): Progressive spasticity and weakness of both lower extremities; pyramidal features in both lower extremities; a positive family history; and exclusion of other diseases (8). The study was approved by the Ethics Committee of Xiangya Hospital, Central South University (Changsha, China). The family members provided written informed consent prior to undergoing a personal interview and a complete neurological examination.

The proband (III5; male, 36 years old) was selected as he was the first case brought to our attention, and had the symptoms of spasticity, weakness and ataxia of both lower extremities, which had persisted for over six years. The patient began to have these symptoms, along with a choking cough following drinking, >six years ago and the symptoms gradually became aggravated. A physical examination revealed poor memory and calculation ability, trouble with speaking clearly, bilateral upper-eyelid contracture, horizontal nystagmus of both eyes, normal muscle power in the upper extremities, inflexibility in alternating movement tests, a lower extremity muscle force of grade 5 (MRC scale) (9), muscle hypertonia, tendon hyper-reflexia and bilateral positive pathological reflexes. The patient was unable to finish the coordination movement examination and had a scissor gait, but he had no sensory abnormalities, muscular atrophy or arched feet. No Kayser-Fleischer ring was noted in the eyes. Chromosome examination revealed a 46, XY karyotype, and brain magnetic resonance imaging (MRI) showed mild atrophy of the cerebellar hemispheres and upper cervical spinal cord. Blood biochemistry tests (triiodothyronine, thyroxine and thyroid stimulating hormone) showed no abnormality. The age of onset in the remaining probands was as follows: I2, 50 years; II2, 42 years; III2, 30 years; III3, 30 years; III7, 27 years; and IV4, 15 years. The age of onset became younger and the symptoms more aggravated in successive generations. The clinical features of three family members are shown in Table I.

### Extraction of genomic DNA (gDNA)

Peripheral venous blood samples (5 ml) were drawn from the patients. Two healthy volunteers outside of the family (N1 and N2) were included as healthy controls. gDNA was extracted by standard phenol-chloroform methods (10). III3 and IV4 did not consent to DNA extraction.

### Polymerase chain reaction (PCR) expansion of Machado-Joseph disease 1 (MJD1) gene trinucleotide repeat fragments

Since genetic anticipation was observed in this family, and pseudo-exophthalmos caused by upper-eyelid retraction is a specific manifestation of hereditary spinocerebellar ataxia type 3 (SCA3)/MJD (7), the MJD1 gene trinucleotide sequences were amplified to detect any mutations. MJD1 gene primers were designed according to the literature (11,12) and were synthesized by Sagon Company (Shanghai, China). The PCR system comprised 200 μmol/l deoxynucleotide triphosphates (Roche Diagnostics GmbH, Nonnenwald, Germany), 0.2 μmol/l of each primer, 50 ng gDNA, 1 unit Taq polymerase (GE Healthcare Life Sciences, Chalfont, UK) and 1 μl 10X PCR buffer (100 mM Tris-HCl, pH 8.5; 500 mM KCl, 1.5% Triton X-100). Deionized water was added to a total volume of 10 μl. A two-phase cycle PCR was used. Firstly, the sample was predenatured at 95°C for 5 min. In the first-phase cycle the samples were denatured at 95°C for 1 min. The initial annealing temperature was 62°C for 1 min, which was decreased by 1°C for each cycle, and extension was performed at 72°C for 2 min, for a total of 10 cycles. In the second-phase cycle, the samples were denatured at 95°C for 1 min, annealed at 52°C for 1 min and extended at 72°C for 2 min, for a total of 25 cycles. The final step was extension at 72°C for 10 min.

### PCR product detection

A 2-μl sample of PCR product was taken and mixed with 2X denaturing buffer (95% formamide, 20 mM EDTA, 0.05% bromophenol blue, 0.05% xylene cyanol). The PCR product was denatured at 99°C for 10 min and cooled rapidly in an ice-bath, the PCR product was then electrophoretically separated in an 8% polyacrylamide gel containing 7 mol/l urea (the electrophoresis buffer was 0.5X TBE; 44.5 Tris-HCl, 44.5 mM boric acid, 1 mM EDTA, pH 8.0). Following 30 min of electrophoresis at <300 V, electrophoresis at 300 V was applied for 4 h. Silver staining and photographic analysis were then carried out. The PCR products of abnormal bands were purified, cloned into the pGEM^®^-T Easy vector (Promega Corp, Madison, WI, USA) and sequenced, and the number of trinucleotide CAG repeats was directly read out from an ABI Prism^®^377 DNA sequencer (Applied Biosystems, Foster City, CA, USA).

## Results

**Figure 2:**
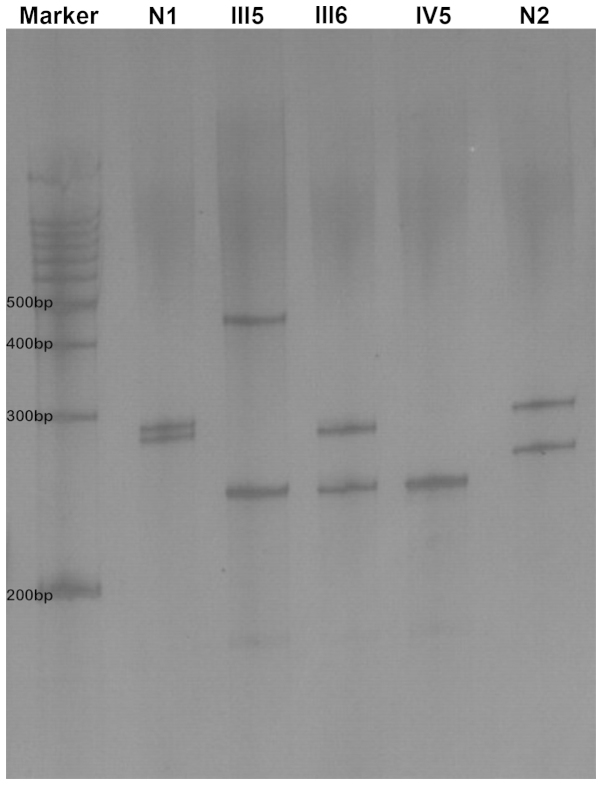
Polymerase chain reaction-amplified CAG repeat in this pedigree.

**Figure 3:**
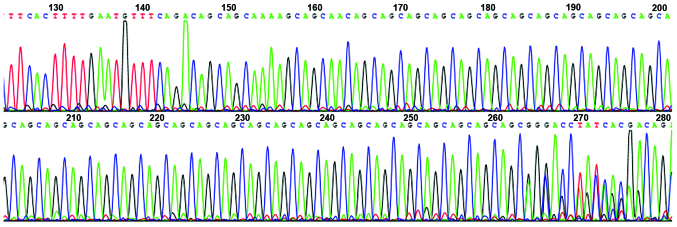
Sequencing diagram of the Machado-Joseph disease 1 gene of the proband.

Proband III5 was found to be a heterozygote for an MJD1 gene mutation, and the CAG repeat number of the mutant allele was 80. The CAG repeat numbers of the wife of the proband (III6) and the son (IV5) were 26 and 15, respectively. All of the patients were heterozygotes. The CAG number of the normal controls was 29 (Figs. 2 and 3).

## Discussion

SCA3/MJD and HSP are common genetic diseases in the clinic and their disease spectrum shows mutual overlaps. The main clinical manifestation of SCA3/MJD is cerebellar ataxia, while other features include pyramidal signs, extrapyramidal symptoms, muscle atrophy, peripheral neuropathy and dementia. It has been reported that SCA3/MJD has several clinical variants. Clinical manifestations include dystonia musculorum deformans, Parkinson’s disease or spastic paraplegia without ataxia (13–16).

The clinical manifestation of the family studied in the present investigation was spastic paraplegia but another significant feature was genetic anticipation. Mild atrophy was present in the cervical spinal cord, as observed using MRI, and the patients exhibited eyelid contracture and horizontal nystagmus. The most well-known disease clinically featuring dynamic mutation is SCA3/MJD, so MJD1 mutation detection was performed. This gave a genetic diagnosis of SCA3/MJD. It was not possible to obtain the DNA from patients III3 and IV4; therefore the dynamic mutation of the MJD1 gene of the family could not be verified at the DNA level. Genetic diagnosis was performed, however, for patient IV5, who was too young to have reached the age-segment of incidence, confirming that the patient did not carry the pathogenic gene.

The cause of clinical variance in patients with SCA is not clear. The larger the CAG repeat number, the earlier the age of onset. Furthermore, the CAG repeat number is, to some extent, associated with the severity of the disease. The more serious the disease, the more impact it is likely to have on the family. The molecular mechanism of genetic anticipation is intergenerational unstable amplification of CAG repeat sequences. There is a trend that the CAG repeat number increases with generation, and the age-at-onset decreases in successive generations. The age of onset is inversely correlated with the CAG repeat number. Maruyama *et al* (17) reported that, in 89 to 100% of patients with SCA3/MJD, dynamic mutations of CAG trinucleotide repeats existed, and the repeat number was 61 to 84. The normal CAG repeat number was 14 to 37. In addition to the CAG repeat number, the phenotype of the disease was affected by certain regulating factors, such as regulating genes, the change in polymorphism within and on either side of the gene loci, genetic imprinting and environmental factors (17,18).

SCA has numerous subtypes with complex and changeable phenotypes. Atypical SCA3/MJD can be manifested as spastic paraplegia without evident ataxia. For those families with HSP involving the nervous system and showing genetic anticipation in the clinic, an MJD1 genetic diagnosis should be considered to compensate for the insufficient diagnosis of HSP. This would promote the prepotency health care and, therefore, gradually reduce the incidence of the disease.

## Figures and Tables

**Table I:** Clinical characteristics of three patients in this pedigree.

Clinical characteristic	IV4	III3	III5
Gender	Male	Male	Male
Age of onset (years)	15	30	29
Course of disease (years)	1	12	7
Bilateral upper extremity weakness	−	−	−
Lower extremity weakness	+	+	+
Bilateral upper-limb tendon reflexes	Normal	Active	Active
Sensory impairment	−	−	−
Bilateral upper-limb muscle strength	Level 5	Level 5	Level 5
Hypertonia of upper limbs	+	++	++
Hoffmann’s sign	−	+	+
Bilateral lower-limb tendon reflexes	Active	Active	Active
Bilateral lower-limb muscle strength	Level 4	Level 4	Level 4
Hypertonia of lower limbs	++	+	+
Feeling of disorder in lower limbs	−	−	−
Ankle clonus	+	+	+
Babinski sign	+	+	+
Gait	Scissor gait	Scissor gait	Scissor gait
Urination obstacles	−	−	−
Dementia	−	−	−
Coordination movement testing	Unable to complete	Unable to complete	Unable to complete
Autonomic nerve dysfunction	−	−	−
Foot deformities	−	−	−
Dysarthria	+	+	−
Exorbitism	−	+	+
Horizontal nystagmus	−	+	+
